# Targeting Porcine Macrophages: The Role of Immune Modulators Against African Swine Fever Virus

**DOI:** 10.3390/vaccines14100904

**Published:** 2026-10-16

**Authors:** Edoardo Candiano, Bruno Tilocca, Giulia Franzoni

**Affiliations:** 1 Department of Veterinary Medicine, University of Sassari, 07100 Sassari, Italy; e.candiano@studenti.uniss.it (E.C.); btilocca@uniss.it (B.T.); 2 Department of Animal Health, Istituto Zooprofilattico Sperimentale della Sardegna, 07100 Sassari, Italy

**Keywords:** ASFV, immunomodulators, TLR-agonists, microbiota, cytokines

## Abstract

African swine fever (ASF) is a highly lethal disease affecting domestic pigs and wild boar, with no safe vaccines available. Macrophages are the primary target cells of African swine fever virus (ASFV), and infection impairs their antiviral functions, thereby facilitating efficient viral replication within these cells and rapid dissemination in the host. This review analyzes host-directed immunomodulatory strategies to functionally reprogram macrophages and restore their defenses. We summarize several classes of immunomodulants, including bacteria, Toll-like receptor (TLR-) agonists, recombinant cytokines, and natural compounds. First, we report recent work on a heat-inactivated bacterium isolated from warthog microbiota: *Rothia nasimurium*, which polarizes macrophages toward an antiviral phenotype and restricts ASFV replication *in vitro*. However, as a live-attenuated vaccine (LAV) adjuvant *in vivo*, it compromises protection by limiting LAV replication. On the contrary, extracellular TLR-2 and TLR-4 ligands induce pro-inflammatory polarization without reducing replication, suggesting that they can safely enhance the poor immunogenicity of inactivated or highly attenuated vaccine candidates. Conversely, intracellular TLR-3, TLR-7/8, and TLR-9 agonists induce type I interferon pathways, and other studies have shown that type I IFNs can restrict ASFV replication. However, direct experimental studies investigating these synthetic ligands against ASFV are currently lacking. Finally, we report that natural compounds like tetrandrine, berbamine, and fangchinoline are able to inhibit ASFV replication *in vitro*, but they can also modulate the macrophage’s immune responsiveness, preventing hyper-inflammation. Targeted immunomodulation may be fundamental to designing effective strategies against ASF. A fine-tuned orchestration of host immunity is required to control viral replication without compromising vaccine efficacy, contributing to the development of safe vaccines or alternative therapeutic tools for this fatal hemorrhagic disease.

## 1. Introduction on African Swine Fever Virus and Literature Search Strategy

African swine fever (ASF) is a highly lethal haemorrhagic viral disease affecting both domestic pigs and wild boar [1,2]. Since its first description in Kenya in 1921 [3], it has been recognized as a severe transboundary threat with a devastating impact on the global swine industry [1,2].

The etiological agent is the African swine fever virus (ASFV), a large enveloped double-stranded DNA virus, belonging to the genus *Asfivirus* within the *Asfarviridae* family [4,5]. Its genome ranges from 170 to 194 kbp and encodes more than open reading frames (ORFs), many of them coding for proteins involved in host range, evasion of immune defenses, and viral pathogenesis [5,6]. As of today, 24 genotypes are accounted for, based on the partial sequence of the *B646L* gene [4]. All genotypes have historically been restricted to Africa, whereas genotype I was initially introduced into Europe in the late 1950s and strains belonging to genotypes I and II have circulated in Asia and Europe in the last 20 years [1,2,7]. More recently, highly virulent ASFV strains containing genetic elements of both genotypes I and II (Recombinant ASFV Genotype I/II Strain) were detected in China and in Vietnam [8,9].

The virus has long persisted in Africa, where it was maintained through a sylvatic cycle involving warthogs and soft ticks of the genus *Ornithodoros* [10]. Outside Africa, ASF spread globally via major historical waves. The first wave (1960–1995) was caused by a genotype I isolate and hit the Iberian Peninsula and other countries in Western Europe [2]. In detail, genotype I was introduced into Portugal in 1957 and then it quickly spread to Spain and other Western European countries. It was successfully eradicated in these countries in 1995, except for Sardinia, where it circulated for several decades [2]. The second wave started in 2007 and was caused by a genotype II strain, which entered Georgia, spreading rapidly through the Caucasus, Russia, and Eastern Europe [11,12]. In August 2018, ASF emerged in China [13] and further spread in Asia, reaching Oceania in 2020 [11]. In 2021, outbreaks were also reported in the Americas [11]. Recently, both genotype II and recombinant ASFV Genotype I/II strains are circulating in China and Vietnam [8,9,14].

Both domestic pigs and wild pigs are highly susceptible to ASFV, and infection with virulent isolates often results in death within a few days [1,15,16]. Contrarily, African wild suids, including warthogs and bushpigs, are naturally resistant to the pathogenic effects of ASFV. Upon infection, these suids develop viraemia but remain clinically healthy [17,18,19]. It was suggested that host genetic factors may reduce over-activation of potentially harmful responses to ASFV, and the reduced clinical signs may also be linked to host tolerance [17].

The disease is subject to compulsory notification and nowadays control relies on rigorous biosecurity measures, culling affected herds, establishing restriction zones, and intensive surveillance [20]. The lack of available vaccines or treatments is hindering the global efforts to control the disease [21]. To date, experimental prototypes of inactivated, subunit, or DNA vaccines have demonstrated safety, but failed to demonstrate the required protective efficacy against the disease. In addition, substantial challenges have arisen in developing live-attenuated vaccines (LAVs) derived from naturally attenuated or genetically modified isolates that maintain a proper balance between safety and efficacy [22,23,24].

One of the main gaps in the rational design of effective and safe vaccines is the limited understanding of the immune-pathogenic mechanisms underlying ASFV infection. The virus mainly targets porcine macrophages, and virulent strains have developed several mechanisms to evade innate immunity, interfering with the IFN response, apoptosis, and antigen presentation [25,26]. Moreover, the replication of highly virulent strains leads to an exacerbated cytokine response (‘cytokine storm’), with subsequent tissue damage, lymphopenia, and widespread hemorrhages [26]. Overall, a better understanding of the complex, multifaceted host–pathogen interactions would benefit the development of safe and effective vaccines [25,26].

A targeted modulation of the immune system is fundamental to designing therapeutic strategies; thus, the role of immunomodulators against ASFV is a research area that deserves further investigation. In this review, we summarized several molecules and their potential application against ASFV, spanning bacteria, Toll-like receptor agonists, recombinant cytokines, and natural anti-inflammatory compounds.

The present narrative review examines relevant literature through a comprehensive search on scientifically recognized, publicly available databases. Here, the literature entries, including abstracts, were collected from PubMed, Web of Science, and Scopus. Such databases were independently queried with the following keywords: ‘African swine fever virus’, ‘ASFV’, ‘immunomodulators’, ‘gut microbiota’, ‘vaccine adjuvants’, ‘Toll-like receptor agonist’, ‘recombinant cytokines’, and ‘natural compounds’. Retrieved results were manually inspected and categorized into specific groups, namely whole bacteria, Toll-like receptor (TLR) agonists, recombinant cytokines, and natural compounds with anti-ASFV activity. Following duplicate removal, a total of 102 articles were assessed and selected for this review.

## 2. Impact of the Microbiota on the Host Response and Its Role in Selecting Bacterial Immunomodulators

The gut microbiota plays a pivotal role in shaping the development and function of the porcine immune system, acting through a complex network of microbial, metabolic, and host-driven interactions. Early colonization of the porcine digestive system establishes the baseline for mucosal and systemic immunity, with commensal bacteria continuously stimulating epithelial and immune cells through pattern recognition receptors, primarily TLRs and NOD-like receptors, thereby calibrating cytokine responses and promoting immune tolerance [27,28]. Alteration of the microbial balance around weaning (i.e., one of the most immunologically vulnerable phases in pig production) contributes to increased intestinal permeability, heightened inflammatory signaling, and impaired mucosal defenses. Microbial metabolites, particularly short-chain fatty acids, further modulate immune homeostasis by enhancing epithelial barrier integrity, regulating NF-κB activity, and promoting regulatory T-cell differentiation. Besides host-associated factors, environmental factors such as diet act as major determinants of the complex network of interactions featuring the super-organism. In this perspective, diet composition and functional feed additives (probiotics, prebiotics, postbiotics) shape microbial communities and influence IgA secretion, antimicrobial peptide expression, and mucus layer function [27,28]. Emerging evidence also highlights the interplay between host genetics and microbiota composition in determining immune competence, suggesting that microbial profiles may contribute to disease resistance and inflammatory phenotypes. Overall, the porcine microbiota acts as a dynamic immunological organ whose modulation offers promising avenues for improving pig health, resilience, and productivity [27,28,29].

Recent work has begun to clarify how the porcine gut microbiota may influence susceptibility or resistance to African swine fever (ASF) [30]. In detail, experimental infection studies in domestic pigs show that ASFV profoundly reshapes the intestinal microbiome, with a reduction in richness (expressed as ACE, Chao1) and a marked loss of SCFA-producing taxa such as *Ruminococcaceae, Roseburia*, and *Blautia*, alongside the expansion of *Proteobacteria* and *Spirochaetes*. Moreover, functional prediction indicates the down-regulation of multiple immune-related pathways, including those involved in immune signaling, barrier maintenance, and energy metabolism, thus supporting a link between ASFV-driven dysbiosis, impaired mucosal and systemic immunity, and exacerbated systemic viral pathology [30]. Complementary evidence comes from fecal microbiota transplantation (FMT) experiments in which piglets receiving feces from naturally resistant warthogs showed increased resistance to ASFV, associated with enhanced mucosal immunity and higher circulating IgA, as well as the modulation of key cytokines such as IFN-γ and TNF-α [31]. Indeed, warthogs employed in the study harbor a microbiota enriched in fiber-fermenting and SCFA-producing communities, associated with stronger epithelial integrity and a more regulated inflammatory tone. Altogether, these studies suggest that specific microbiota configurations, particularly those enriched in SCFA-producing and immunostimulatory communities, may contribute to a more effective antiviral response, whereas ASFV-induced dysbiosis could exacerbate disease severity; they also open the way to microbiota-targeted strategies (e.g., FMT, probiotics, diet) as adjuncts to ASF control in swine populations [31]. Although the literature is still limited, the convergence of infection-model, comparative, and FMT evidence suggests that the microbiota may be one of the determinants of ASF outcome, opening avenues for microbiota-targeted interventions in swine health management.

A recent study also analyzed the diverse response of specific pathogen-free (SPF) and farm-raised Large White domestic pigs to a moderately virulent ASFV isolate (Estonia 2014) [32]. The two groups presented substantial differences in gut microbiome composition, alongside distinct basal inflammatory and antiviral transcriptomic profiles. Consequently, the two groups responded differently to virus infection. SPF pigs presented a milder and shorter clinical disease with a 100% survival rate, whereas farm pigs showed a severe, protracted disease with only a 50% survival rate. In addition, farm pigs exhibited a stronger pro-inflammatory response characterized by a higher production of pro-inflammatory cytokines (IL-1α, IL-2, IL-6, IL-8, and IL-18), whereas the response of SPF pigs was distinguished by elevated levels of the anti-inflammatory cytokine IL-1Ra [32]. Future studies should investigate whether a controlled microbiota could contribute to a more balanced host immune response and enhanced resilience against moderately virulent ASFV strains, preventing the onset of a lethal cytokine storm.

As stated above, ASFV mainly targets porcine macrophages, and virulent strains have developed several mechanisms to evade innate immunity to efficiently replicate within these cells [25]. ASFV infection of macrophages leads to a reduced expression of CD14 and CD16, with a potential impairment of their antimicrobial and antiviral activities and the down-regulation of several antiviral genes; this suggests that the infection progressively impairs macrophage defenses, thereby allowing viral replication [25,33]. The use of whole bacteria or their derivatives as immunomodulators could help macrophages reprogram their functions to restore both antiviral and phagocytic capacities. Some interesting attempts have recently been made. Tort-Miró and collaborators (2025) analyzed the immunomodulatory properties of *Rothia nasimurium*, a bacterium isolated from warthog (*Phacochoerus africanus*) fecal microbiota [34]. The authors demonstrated that heat-inactivated *R. nasimurium* triggered a robust activation of the pig innate immune system, characterized by enhanced phagocytic activity, activation of the inflammasome, and production of IL-1β and TNF-α by porcine alveolar macrophages (PAM). In addition, a strong TNF-α release was detected in response to ASFV infection several days after the initial bacterial stimulation, accompanied by a reduction in ASFV replication kinetics. These findings suggested that *R. nasimurium* might enhance animal resilience to ASFV through the fine-tuning of innate immune cell responsiveness [34]. Following these promising *in vitro* results, the same research group investigated the potential use of this bacterial immunomodulator as an adjuvant for the candidate vaccine ASF BA71ΔCD2 LAV. Unexpectedly, the use of the inactivated bacterium decreased both humoral and cellular responses induced by BA71ΔCD2, with subsequent lower protection against challenge with a virulent ASFV isolate. These unexpected results were likely due to the antiviral state of macrophages triggered by the adjuvant, which limited LAV replication in the host and, consequently, its ability to trigger the development of a protective immune response. Therefore, the authors suggested that the association of bacterial adjuvants with ASFV-based LAVs would require a fine-tuned macrophage activation, in order to increase functionality without excessively inhibiting LAV replication [35].

**Figure 1:**
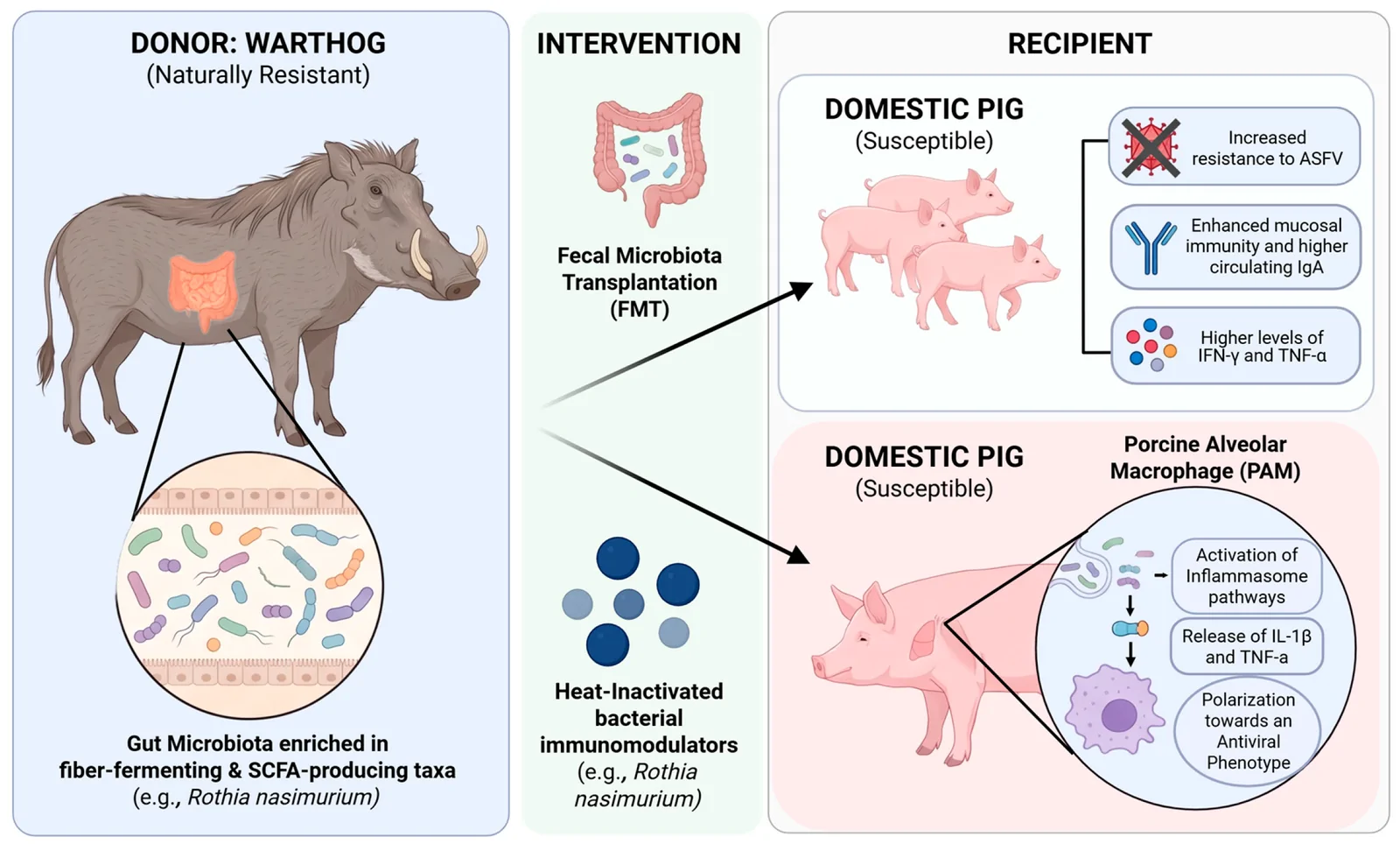
Overview of microbiota-derived interventions against ASF. The impact of warthog fecal microbiota transplantation in shaping porcine immune responses is presented, alongside the effects of heat-inactivated *Rothia nasimurium* on macrophage polarization against ASFV. Image created with BioRender.com (accessed on 12 October 2026).

A schematic overview of microbiota-derived interventions in shaping porcine immune responses and macrophage polarization against ASF is provided in Figure 1. The most important findings of *R. nasimurium*’s impact on porcine macrophages and its potential use against ASFV are summarized in Table 1.

## 3. TLR Ligands as Immunomodulators: Opportunities or Pro-Viral Effects?

### 3.1. TLR-s and Their Ligands

Another important family of immunomodulatory molecules is represented by Toll-like receptor (TLR) agonists. TLRs are a family of pattern recognition receptors (PRRs) that play a crucial role in initiating host immune defenses against invading pathogens. These receptors specifically recognize highly conserved components essential for microbial survival, termed pathogen-associated molecular patterns (PAMPs), and endogenous elements released during tissue injury, known as danger-associated molecular patterns (DAMPs) [36,37]. Based on their cellular localization, TLRs are broadly divided into two main subfamilies: extracellular receptors situated on the cell membrane (including TLR-1, TLR-2, TLR-4, TLR-5, and TLR-6), which predominantly sense microbial surface components like lipopolysaccharides or lipopeptides, and intracellular/endosomal receptors (such as TLR-3, TLR-7, TLR-8, and TLR-9), which are specialized in detecting viral and bacterial nucleic acids [36,37].

The binding between a TLR and its ligand triggers a complex cascade of intracellular signaling pathways. This network activates the inflammasome, with subsequent induction of a localized inflammatory response and production of type I interferons (IFNs), which limit pathogen progression [38,39]. Since TLR ligands target host pathways rather than the virus itself, they represent innovative prophylactic and therapeutic options [38]. This host-directed mechanism provides a broad spectrum of activity and avoids the risk of resistance. Moreover, synthetic TLR agonists serve as effective adjuvants in vaccination. By delivering a strong danger signal, they stimulate host immunity and promote a robust, long-lasting adaptive immune response [37]. However, tight regulation of these cascades is essential: while a baseline activation is required for pathogen clearance, uncontrolled signaling must be avoided to prevent exacerbated and pathological damage [39].

### 3.2. Agonists of TLR-2 and Their Potential Use Against ASFV: Evaluation of Mag-Pam2Cys_P48

TLR-2 is a surface receptor that predominantly senses microbial surface components and forms heterodimers with both TLR-1 and TLR-6. Triacyl-lipoproteins are recognized by TLR-2 in combination with TLR-1, whereas diacyl-lipoproteins activate TLR-2 in combination with TLR-6 [38]. Previous studies showed that TLR-2 agonists, such as the *Mycoplasma fermentans*-derived lipopeptide MALP-2 and its synthetic analog Pam2Cys, have significant therapeutic and prophylactic potential against both bacterial and viral pathogens by activating host innate immunity [40,41,42]. Intranasal administration of these ligands, including the pegylated variant PEG-Pam2Cys or novel synthetic TLR-2/TLR-6 agonists (INNA-051), stimulates rapid immune cell recruitment in the injection site and establishes a protective antiviral environment. This approach successfully restricted replication of both influenza A (in mice) and SARS-CoV-2 (in ferrets) [42,43].

Based on these antiviral findings, our research group investigated the immunomodulatory potential of a chemically synthesized diacylated lipopeptide: Mag-Pam2Cys_P48. Our data showed that the stimulation of porcine monocyte-derived macrophages (moMΦ) with this synthetic TLR-2 agonist resulted in a pro-inflammatory and antimicrobial polarization, characterized by the up-regulation of activation markers (MHC class I, MHC class II DR, and CD25), enhanced phagocytic capacity, release of pro-inflammatory cytokines, and enhanced expression of multiple interferon-alpha (IFN-α) subtypes [44]. Consequently, we evaluated the capacity of Mag-Pam2Cys_P48 to modulate host macrophage defenses against two genotype I ASFV strains of diverse virulence: the attenuated NH/P68 and the highly virulent 26544/OG10. Porcine moMΦ pre-treated with Mag-Pam2Cys_P48 exhibited a pro-inflammatory phenotype, with significantly higher release of pro-inflammatory IL-1α and IL-1β upon ASFV infection than mock-infected controls. Surprisingly, this pre-activation failed to restrict the replication kinetics of either strain. On the contrary, the highly virulent ASFV 26544/OG10 isolate replicated even slightly more efficiently in macrophages pre-treated with this TLR-2 ligand [45]. These data highlighted the limited applicability of these TLR-2 agonists as prophylactic or immune-potentiating agents against virulent ASFV; however, they have raised novel questions, such as whether virulent ASFV strains replicate more efficiently within a pro-inflammatory microenvironment. On the other hand, Mag-Pam2Cys_P48 down-regulated the expression of cGAS and TLR-3, -7, -8, and -9 [45], which might impair the ability of porcine moMΦ to fight ASFV. Further studies should be carried out to investigate these mechanistic aspects.

### 3.3. Agonists of Other Extracellular TLR-s and Their Potential Use Against ASFV

Other extracellular receptors are TLR-4 and TLR-5. TLR-4 ligands include lipopolysaccharide (LPS) from Gram-negative bacteria, monophosphoryl lipid A from Gram-negative bacteria, Fimbriae H protein (FimH) from *Escherichia coli*, synthetic compounds Aminoalkyl glucosaminide phosphates (AGPs), fusion (F) protein of respiratory syncytial virus, envelope protein from mouse mammary tumor virus, heat shock proteins, hyaluronic acid, and beta defensin 2 [38,46]. TLR-5 ligands include, instead, bacterial flagella proteins [38,47]. The recognition of these compounds by TLR-4 or TLR-5, respectively, activates MyD88 and NF-kb, with subsequent release of pro-inflammatory cytokines and polarization of macrophages towards an anti-microbial phenotype [38].

LPS is a strong immunomodulatory agent and in pigs LPS triggered production of pro-inflammatory cytokines (IL-1β, IL-8, IL-12, and TNF), enhanced phagocytic activity, and up-regulation of activation markers (MHC II, CD40, CD80/86) by PAM and moMΦ [48,49,50,51]. Nevertheless, LPS possesses strong toxicity; therefore, its clinical use as a vaccine adjuvant is extremely limited [38]. Studies carried out in mice showed that other TLR-4 agonists, such as monophosphoryl lipid A and FimH, are potent immunomodulators that enhance both systemic and mucosal immunity, making them promising vaccine adjuvants [52,53,54]. TLR-5 ligands were also tested using rodent models. Previous studies reported that the TLR-5 ligand flagellin can act as a potent immunostimulant and has demonstrated strong immune-stimulatory effects against several pathogens [47,55,56]. Nevertheless, to date, few studies on these TLR ligands have been performed in large animals.

In pigs, the mucosal adjuvant activity of two extracellular TLR ligands, namely flagellin from *Vibrio vulnificus* (TLR-5) and mHsp70 from *Mycobacterium tuberculosis* (TLR-4), was compared by Lu et al. (2021) [57]. Researchers used these TLR ligands as adjuvants for recombinant Adenoviral (rAd) vectors expressing ASFV antigens CD2v, p30, and p54 [57]. Both adjuvanted vectors, via intranasal immunization, induced enhanced systemic and mucosal immune responses in pigs, including antigen-specific IgG and IgA antibodies and Th1/Th2 cytokine production. However, mHsp70 generally elicited stronger mucosal responses than FlaB flagellin, with higher IgA production and antigen-specific release of IL-4, IL-10 and IFN-α by PAM. Therefore, the authors proposed the mHsp70 fusion rAd vector as a promising platform for mucosal ASFV vaccine development [57].

Nevertheless, recent findings suggested that TLR-4 activation enhances ASFV replication, as observed for TLR-2 agonists, particularly regarding virulent isolates. Our research group previously investigated the impact of lipopolysaccharide (LPS), a well-known pro-inflammatory TLR-4 ligand, on ASFV replication at 24 h post-infection. The stimulation of porcine moMΦ with 100 ng/mL of LPS drastically impaired the replication kinetics of the non-virulent BA71V strain, whereas this pre-treatment failed to affect the replication efficiency of the highly virulent 22653/14 isolate [58]. In a more recent work, Chen and collaborators reported that a TLR-4 inhibitor (resatorvid) inhibited ASFV replication in PAM, implying that replication of virulent strains may be dependent on the TLR-4 signaling pathway [59]. These data highlighted the limited applicability of TLR-4 agonists as prophylactic or immune-potentiating agents against virulent ASFV; however, they can safely enhance the poor immunogenicity of inactivated or highly attenuated vaccine candidates.

### 3.4. Agonists of TLR-3, 7, 8, 9 and Their Potential Use Against ASFV

TLR-3, TLR-7, TLR-8, and TLR-9 are intracellular/endosomal receptors, which are specialized in detecting viral and bacterial nucleic acids [36,37].

TLR-3 recognizes double-stranded RNA, which triggers the MyD88-independent signaling pathways, through the adapter molecule Toll/IL-1R (TIR) domain-containing adapter-inducing interferon-β (TRIF). Its stimulation leads to the activation of IFN regulatory factor 3 (IRF3), which results in an up-regulation of type I IFN expression [38,60].

TLR-3 ligands include polyinosine–polycytidylic acid (poly(I:C)), poly (I:C) complexed with poly-L-lysine and carboxymethylcellulose (Poly-ICLC), liposome-encapsulated Poly-ICLC (LE Poly-ICLC), and polyadenylic–polyuridylic acid (poly(A:U)) [38,61,62]. These molecules are synthetic analogs of double-stranded RNA that bind to TLR-3 in the endosome, leading to type I IFN expression. When used as vaccine adjuvants, they enhance the development of both humoral and cellular immune responses and provide clinical protection in commercial and experimental vaccines [61,62].

In pigs, poly(I:C) has been shown to induce the mRNA expression of MHC class II, CD80/CD86, IL-12p35, IFN-γ, TLR-5, and TLR-9 in myeloid antigen-presenting cells [63]. Additionally, a more recent study demonstrated that it enhances the release of TNF-α by pDCs, as well as the release of IL-12 and IFN-α by CD1+, cDCs, and pDCs [64]. Furthermore, poly(I:C) triggers enhanced expression of several ISGs in PAMs [65]. Nevertheless, direct experimental studies investigating poly(I:C) against ASFV are currently lacking.

TLR-7 and TLR-8 recognize single-stranded RNA fragments located in endosomes; thus, their activation is frequent during viral infection [38]. They possess a high degree of sequence homology and functional similarity. The recognition of single-stranded RNA by these TLRs triggers the MyD88-dependent signaling pathways, leading to the activation of NF-κB and the release of pro-inflammatory cytokines [38,66,67]. TLR-7 is predominantly expressed in pDC and B cells, whereas TLR-8 is more strongly expressed in myeloid dendritic cells and monocytes, and to a lesser extent in pDCs [67].

TLR-7/8 ligands include synthetic tricyclic derivatives belonging to the imidazo[4,5-c]quinoline series, such as resiquimod (R-848), imiquimod, gardiquimod [67], and pteridinone GS-9620 [68]. Most of the studies were carried out on human viruses [38,61,69], although some compounds have also been tested in pigs [61]. In particular, previous studies reported that imiquimod and gardiquimod markedly activate both porcine TLR-7 and TLR-8, which are located mainly within the endoplasmic reticulum; thus, their activation is likely dependent on endosomal/lysosomal acidification [70]. TLR-9 recognizes CpG and synthetic oligonucleotides displaying non-methylated CpG, which activate the MyD88-dependent signaling pathways, leading to the subsequent activation of NF-κB and the release of pro-inflammatory cytokines [38]. In pigs, TLR-9 expression has been reported in pDCs, cDCs, and B cells [71,72], as well as in macrophages and epithelial cells [73]. Like TLR-7 and TLR-8, TLR-9 is located mainly within the endoplasmic reticulum; thus, its activation is dependent on endosomal/lysosomal acidification [74].

TLR-9 ligands include CpG ODNs (cytidine-phosphate-guanosine oligodeoxynucleotides), which enhance immune responses against several intracellular pathogens in a mouse model [38]. Many of these compounds were also tested in pigs [61,75,76,77] and Kamstrup and colleagues reported that several CpG-ODNs triggered the release of pro-inflammatory cytokines (IL-6, IL-12, and TNF-α) by porcine peripheral blood mononuclear cells (PBMCs) [75]. As adjuvants, CpG ODNs enhance both humoral and cell-mediated immune responses, thereby improving protective efficacy across several swine vaccines, such as PRRSV and swine influenza [61,76,77].

However, many of these TLR ligands have been tested against human and other mammalian viruses, but no studies have investigated their antiviral and immunomodulatory effects against ASFV.

ASFV is sensed mainly by the cGAS-STING pathway, although both the RNA sensor TLR-3 and the DNA sensor TLR-9 contribute to its recognition [78]. The virus employs multiple strategies to block these intracellular TLR-s, ensuring efficient viral replication and transmission. In detail, ASFV encodes two inhibitors of TLR-3: A276R and I329L [79,80]. In addition, recent studies have revealed that the inhibitory effect of ASFV I329L also extends to TLR-9 and other TLR-s (TLR-4, TLR-5, and TLR-8) [81]. In agreement, Portugal and collaborators observed that ASFV inhibited type I IFN production in macrophages stimulated with the TLR-3 agonist poly(I:C) [65]. Considering that the virus possesses mechanisms to inhibit these TLR- pathways, their targeting using synthetic ligands could represent a useful strategy. Future studies should further investigate the effects of such ligands on the host’s capacity to respond to ASFV.

**Figure 2:**
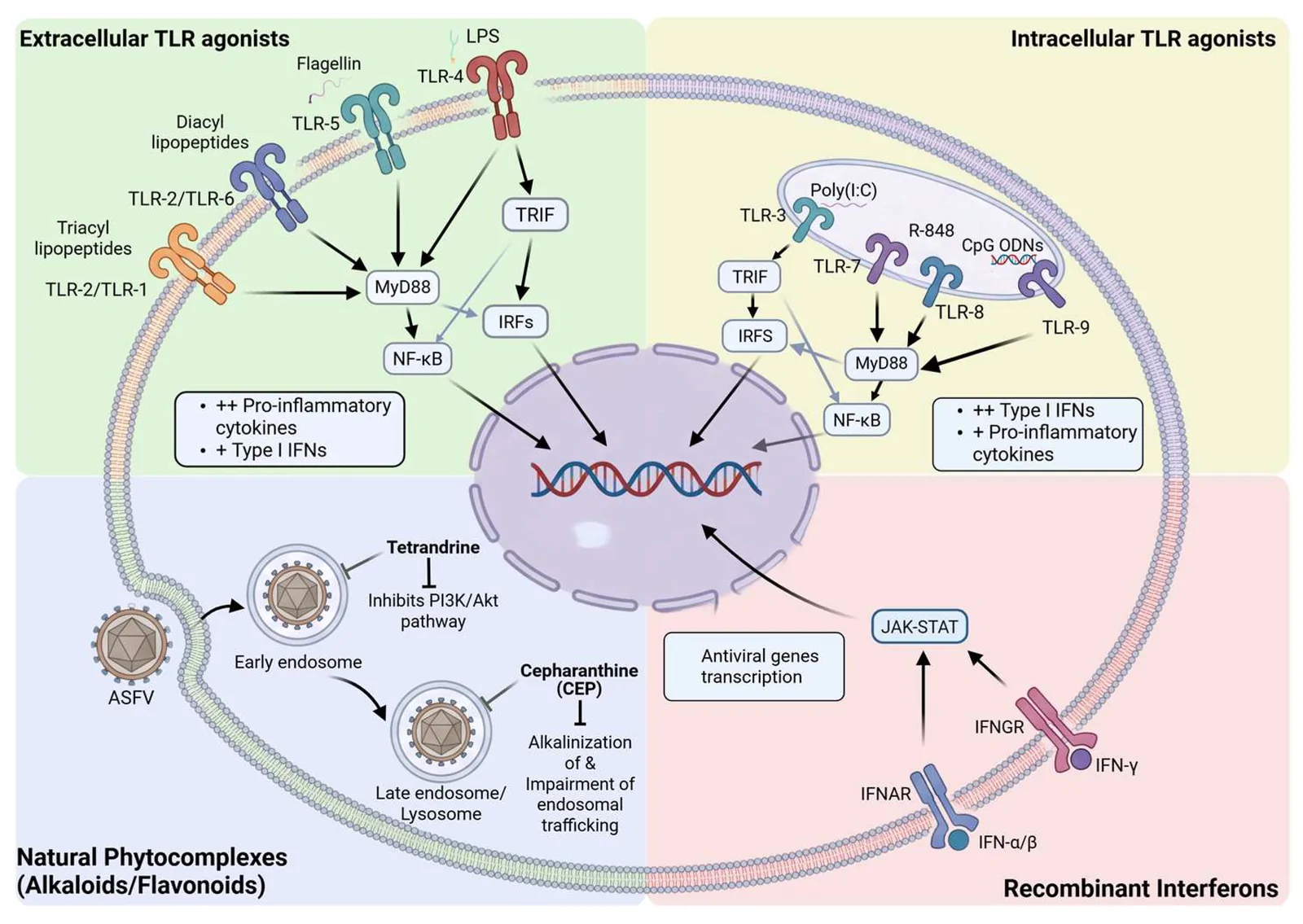
Macrophage-targeted immunomodulatory interventions against ASFV. A schematic overview of macrophage-targeted immunomodulatory interventions against ASFV is provided, including extracellular and intracellular TLR agonists, recombinant cytokines, and natural phytocomplexes. Image created with BioRender.com (accessed on 12 October 2026).

The most important findings of TLR ligands’ impact on porcine macrophages and their potential use against ASFV are summarized in Table 1. A schematic representation of the impact of extracellular and intracellular TLR ligands on porcine macrophages is provided in Figure 2.

## 4. Alternative Immunomodulatory Strategies

### 4.1. Immunomodulators Targeting Internal Cell Signaling Pathways

Another research area focuses on targeting internal cell signaling pathways through recombinant cytokines, natural compounds, and anti-inflammatory phytocomplexes, bypassing direct microbial stimuli. In 2019, Arabyan and collaborators summarized compounds with anti-ASFV properties, including nucleoside analogs, interferons, plant-derived compounds, antibiotics, and small interfering RNA [82]. In our review, we provided a recent up-date of anti-ASFV activity of recombinant cytokines and natural compounds with anti-ASFV activity. The most important findings regarding the impact of these molecules on porcine macrophages, along with their potential application against ASFV, are summarized in Table 1 and a schematic representation is provided in Figure 2.

### 4.2. Recombinant Type I and II Interferons

The adjuvanticity of several recombinant cytokines, including IL-1, IL-2, IL-4, IL-6, IL-12, IL-15, IL-18, IL-19, IFNα, and IFNγ, in pigs was recently reviewed by Charerntantanakul [61]. The author reported that the adjuvanticity of most cytokines depends largely on their dose, form, route, schedule of administration, and the type of co-administered vaccine. Among these cytokines, the only ones tested against ASFV to date are type I and type II IFNs [61].

Interferons (IFNs) are a family of cytokines that play a crucial role in orchestrating the innate immune response during viral infections. These proteins represent the first line of defense in cells during viral challenges [83]. Three distinct classes of IFNs have been described: Type I, Type II, and Type III IFNs, with type I IFNs, especially IFN-α subtypes and IFN-β, exhibiting the strongest antiviral activities [84]. Upon receptor binding, type I IFNs activate the transcription of several interferon-stimulated genes (ISGs), which encode a heterogeneous group of proteins mediating direct antiviral defense and immune regulation [85].

IFN-γ represents the only porcine type II IFN, and it is mainly secreted by CD4+ Th1 cells, NK cells, and CD8+ cytotoxic T cells. IFN-γ exerts its function through binding to the IFN-γ receptor (IFNGR), which activates a downstream signal transduction cascade that leads to the regulation of the expression of several genes [84]. This cytokine promotes inflammation, NK cell activity, and classical macrophage activation. Furthermore, IFN-γ possesses strong antiviral activity; thus, several viruses have evolved multiple strategies to evade its effects [26,84].

ASFV has evolved multiple strategies to counteract and evade the effects of type I and type II IFNs [86,87]. Virulent isolates possess multiple copies of *MGF360* and *MGF530/505* gene families, which suppress the induction of type I IFN by infected macrophages [88]. In addition, virulent isolates, such as the L60 strain, inhibited the expression of several ISGs, such as *ISG15*, *PKR*, and *MX1* [65]. More recent studies showed that the inhibition of the production of type I IFN is mediated mainly by antagonizing the cGAS-STING signaling pathway [89,90].

Direct administration of recombinant porcine type I IFNs overcomes the inhibitory mechanisms of the virus on the cGAS-STING pathway. Recombinant IFN-α and IFN-β proteins bind directly to the cell-surface IFNAR receptor, leading to the activation of the classical JAK-STAT signaling cascade and induction of several ISGs, such as *MX1* and *ISG15* [83]. *In vitro* studies showed that recombinant IFN-α restricted the replication of the highly virulent genotype II ASFV strain SY18 in PAM [87], as well as the genotype I ASFV strains NH/P68 (attenuated) and 22653/14 (highly virulent) in moMΦ, although the latter was affected to a lesser extent [91]. On the contrary, Reis and collaborators observed that recombinant IFN-α (2000 IU/mL) did not inhibit the replication of virulent ASFV isolates (BA71, Georgia 2007/1, and OUR T88/1) in PAM, whereas it significantly reduced the replication of the low-virulent OUR T88/3. In the same study, researchers discovered that the *MGF360* and *MGF505* genes are those that enable ASFV to overcome this antiviral state [88].

Similarly, it has been described that virulent ASFV isolates have developed mechanisms to efficiently replicate within macrophages stimulated with this antiviral molecule [25]. Franzoni et al. previously reported that this recombinant IFN drastically inhibits the replication of the avirulent BA71V strain, but not that of the highly virulent 22653/14 isolate, in moMΦs [58]. In contrast, Fan and colleagues observed that recombinant IFN-γ restricts the replication of the genotype II ASFV strain SY18 in PAMs [87]. The differences between these two studies might be linked to the amount of IFN-γ used to stimulate porcine macrophages (1000 U/mL in the study by Fan and colleagues, versus 100 ng/mL in the work by Franzoni and collaborators) [58,87].

### 4.3. Natural Compounds with Anti-ASFV Activity

Increasing research has shown that natural compounds are widely used in antiviral research because of their broad bioactivity and diverse sources. It was reported that plant-derived compounds such as apigenin, genistein, and genkwanin inhibited ASFV replication *in vitro* [82,92]. It was observed that apigenin possessed a strong, dose-dependent, anti-ASFV activity *in vitro* [93], although this plant-derived flavonoid was chemically unstable and practically insoluble in water. Therefore, in a subsequent study, six commercially available apigenin derivatives (acacetin, apigetrin, genkwanin, rhoifolin, vitexin, and vitexin 2-O-rhamnoside) were screened using the non-virulent BA71V and the monkey cell line VERO, and data revealed that genkwanin possessed a potent anti-ASFV activity [94]. Subsequently, researchers showed that genkwanin also inhibited the replication of the virulent genotype II Armenia/07 ASFV strain in PAM [94]. The same research group observed that another plant-derived compound, named genistein, similarly inhibited ASFV replication *in vitro* by decreasing the synthesis of ASFV viral proteins p32 and p72, thereby reducing viral titres in culture supernatants [92]. This compound inhibited both BA71V replication in VERO and Armenia/07 ASFV replication in PAM [92].

Below, we report recent studies focused on new plant-derived compounds with anti-ASFV activity, where researchers deeply investigated their underlying action mechanisms. Main results are summarized in Table 1. While many of these natural phytocomplexes exert a direct host-directed antiviral activity by targeting cellular entry or intracellular trafficking pathways, they are included in this review due to their specific macrophage-targeted mechanisms. Furthermore, increasing evidence demonstrates that these compounds also possess potent immunomodulatory and anti-inflammatory properties, enabling them to dampen the exacerbated release of pro-inflammatory cytokines in ASFV-infected macrophages.

Chen and collaborators (2023) screened 102 compounds against ASFV and identified 17 compounds with anti-ASFV activity. The selectivity index (SI) of kaempferol, quercetin, naringenin, formononetin, resveratrol, luteolin, sinensetin, licochalcone A, and dihydromyricetin (DHM) was higher than that of genistein (5.59), which was used as a positive control. The compound with higher antiviral activity was DHM [59]. Researchers observed that it inhibited ASFV replication in a dose-dependent manner. In particular, Chen and colleagues observed that 100 µM was non-cytotoxic in PAM and this concentration reduced virus replication, assessed using both viral titration (hemadsorption) and RT-qPCR (expression of B646L mRNA) [59]. In addition, researchers observed that the compound reduced ASFV, suppressing pyroptosis by regulating TLR-4 signaling. As previously stated in this review, inhibition of TLR-4 inhibited ASFV replication in PAM, and indeed this DHM reduced replication of this virus by targeting the TLR-4/MyD88/MAPK/NF-kB pathway [59]. In another study, Jo and colleagues reported that a similar flavonoid compound (myricetin) inhibited ASFV protease [95]. It was speculated that these flavonoid compounds inhibited ASFV replication in a similar manner [59]. Future studies should further investigate the effects of these flavonoid compounds against the virus, expanding on these promising *in vitro* studies with *in vivo* experiments.

Later, Jackman and collaborators (2024) screened 297 compounds against ASFV, using the avirulent BA71V and the monkey cell line VERO, and identified 5 compounds with anti-ASFV activity: paeonol, pinoresinol, curcumin, berbamine, and tetrandrine. The compounds with higher antiviral activity were tetrandrine and berbamine and these were further tested on PAM, with the virulent ASFV strain Arm/07 (genotype II) [96]. Researchers observed that these compounds inhibited ASFV replication, assessed using viral titration (hemadsorption), in a time-dependent manner. They inhibited infection at an early stage, with a reduction in virus yield observed when tetrandrine or berbamine was added during the virus attachment substage, and even more efficiently during the virus internalization substage [96]. These results were in line with those of Zhu and collaborators, who observed that berbamine hydrochloride inhibited replication of highly virulent genotype II ASFV strain GZ201801, in a dose-dependent manner and at an early stage of ASFV infection [97]. In addition, Jackman and colleagues observed that these compounds inhibited cytokine release by ASFV-infected PAM. Finally, tetrandrine exhibited a stronger anti-inflammatory and anti-ASFV effect than berbamine [96]. In another study, it was demonstrated that tetrandrine inhibited ASFV at the internalization stage by blocking macropinocytosis through the inhibition of the *PI3K/Akt* pathway [98].

Su and collaborators (2024) investigated instead the anti-ASFV activity of fangchinoline (FAN), a bis-benzylisoquinoline alkaloid whose antiviral activity against other viruses had previously been reported. Its effects were investigated on PAM using the virulent ASFV GZ201801 (genotype II) [99]. The compound was tested at the maximum concentration of 10 μM, and it showed a strong reduction in viral replication, as evidenced by decreased viral protein (p30) expression, viral B646L genome copy number, viral CP204L mRNA levels, and viral titre at both 24 and 48 hpi. The efficacy of the treatment was dose-dependent and the compound had the strongest effect in the pre-treatment mode. In addition, researchers observed that FAN suppressed ASFV-induced activation of the AKT/mTOR/NF-κB signaling pathway, with reduced expression of IL-1β and TNF-α in ASFV-infected PAM [99].

Finally, Zhu and collaborators (2024) further investigated the anti-ASFV activity of bis-benzylisoquinoline alkaloids (BBAs), focusing on four different compounds: cepharantine (CEP), tetrandrine, fangchinoline, and isotetrandrine. Their effects were investigated on PAM using the virulent ASFV GZ201801 (genotype II). Researchers observed that all tested BBAs inhibited ASFV replication in a dose-dependent manner, reflected by a reduction of B646L and CP204L transcription, p72 expression, and viral titres [100]. Among these compounds, CEP was further investigated, showing antiviral activity at multiple stages of the ASFV replication cycle, particularly during the early stages of infection. CEP accumulated in lysosomes and induced their alkalinization and morphological alterations, impaired endosomal trafficking, and therefore prevented proper transport and uncoating of ASFV, while also suppressing viral DNA synthesis and the formation of viral replication factories. These findings identified the endolysosomal compartment as an important host target for BBAs and highlighted the importance of lysosomal alkalinization in their anti-ASFV activity [100].

In Table 1, for all the immunomodulators, we highlighted the specific macrophage model utilized in each study (PAM vs. moMΦ). PAM and moMΦ possess distinct phenotypes, with diverse basal activation states and surface receptor expressions [101], and thus their antiviral responses can differ. Such biological heterogeneity must be carefully taken into account when interpreting apparently conflicting results across different experimental studies.

**Table 1:** Immunomodulators and their impact on macrophage polarization and ASFV replication.

Immune-Modulators	Impact on Porcine Macrophages	Impact on ASFV Replication	References
Heat-inactivated *Rothia nasimurium*	Enhanced phagocytic activity, activation of the inflammasome, and cytokine production by PAM.	Reduction in ASFV replication kinetics *in vitro*: virulent Georgia2007/1 and attenuated BA71ΔCD2 (evaluated with flow cytometry). *In vivo*, it decreased both humoral and cellular responses induced by BA71ΔCD2, with lower protection against challenge with a virulent ASFV isolate.	[34] [35]
Mag-Pam2Cys_P48 (TLR-2 agonist)	Upregulation of activation markers, enhanced phagocytic capacity, release of pro-inflammatory cytokines, and enhanced expression of multiple IFN-α subtypes by porcine moMΦ.	No restriction of the replication kinetics of both attenuated NH/P68 and virulent ASFV 26544/OG10. The latter replicated slightly more efficiently in porcine moMΦ pre-treated with this TLR-2 ligand. Enhanced release of IL-1α and IL-1β upon ASFV infection compared with mock-infected controls.	[44] [45]
LPS (TLR-4 agonist)	Upregulation of activation markers and strong release of pro-inflammatory cytokines by porcine moMΦ [49,50].	The stimulation of porcine moMΦ with 100 ng/mL of LPS drastically impaired the replication kinetics of the avirulent BA71V strain, but it did not affect the replication of the highly virulent 22653/14 isolate [58]. The TLR-4 inhibitor resatorvid inhibited ASFV replication in PAM, suggesting that replication of virulent strains may be dependent on the TLR-4 signaling pathway [59].	[49] [50] [58] [59]
TLR-3 agonist	Release of type I IFN, induction of several ISGs, including *MX1, PKR, Bst2*, and *ISG15* in porcine moMΦ [65].	Unknown	[65]
Porcine Recombinant IFN-α/IFN-β (Type I IFNs)	Induction of several ISGs in both porcine moMΦ [65] and PAM [87], including *MX1, PKR,* *Bst2*, *ISG15*, *IFIT1, IFITM3, OASL, GBP1, Viperin, IRF-1,* and *CXCL10* [65,83,87].	Recombinant IFN-α restricted the replication of the ASFV strain SY18 in PAM (genotype II) [87]. Recombinant IFN-α restricted the replication of the low-virulent NH/P68 and highly virulent 22653/14 (both genotype I) in porcine moMΦ, although the latter was less affected [91]. Recombinant IFN-α (2000 IU/mL) did not inhibit the replication of virulent ASFV isolates (BA71, Georgia 2007/1, and OUR T88/1) in PAM, whereas it significantly reduced the replication of the low-virulent OUR T88/3 [88].	[65] [83] [87] [88] [91]
Porcine Recombinant IFN-γ (Type II IFNs)	Polarization of porcine macrophages towards a pro-inflammatory phenotype, with up-regulation of MHC I, MHC II, and CD11a in porcine moMΦ [50,102]. Induction of several ISGs, including *MX1,* *Bst2*, *ISG15*, *IFIT1, IFITM3, OASL, PKR, GBP1, Viperin, IRF-1,* and *CXCL10* in PAM [87].	Recombinant IFN-γ (1000 U/mL) restricted the replication of the genotype II ASFV strain SY18 in PAM (MOI 1) [87]. Recombinant IFN-γ (100 ng/mL) restricted the replication of the avirulent BA71V but not the highly virulent 22653/14 (both genotype I) in porcine moMΦ (MOI 1) [58].	[58] [87]
Dihydromyricetin (DHM)	Cytotoxic effect in PAM only at concentrations exceeding 200 IU/mL.	DHM at 100 IU/mL reduced virus replication (MOI 1), assessed using both viral titration (hemadsorption) and RT-qPCR (expression of B646L mRNA). DHM reduced ASFV by regulating TLR-4 signaling.	[59]
Berbamine and tetrandrine	Cytotoxic effect in PAM only at high concentrations: cytotoxic effect in PAM only at concentrations exceeding 50 IU/mL for tetrandrine [96] CC_50_ of berbamine hydrochloride was 27.89 μM [97] CC_50_ of TET in PAM is 12.12 μM [98].	Berbamine hydrochloride inhibited replication of highly virulent genotype II ASFV strain GZ201801 in a dose-dependent manner. The antiviral activity of berbamine hydrochloride was maintained for 48 h, and the compound blocks the early stage of ASFV infection [97]. Both compounds inhibited Arm/07 ASFV replication, assessed using viral titration (hemadsorption) in a time-dependent manner. A reduction in virus yield was observed when tetrandrine or berbamine was added during the virus attachment substage, and even more efficiently during the virus internalization substage [96]. These compounds inhibited cytokine release by ASFV-infected PAM. Tetrandrine exhibited a stronger anti-inflammatory and anti-ASFV effect than berbamine [96]. Tetrandine inhibited replication of ASFV China/2018/AnhuiXCGQ at the internalization stage by blocking macropinocytosis through the inhibition of the PI3K/Akt pathway [98].	[96] [97] [98]
Fangchinoline (FAN)	Cytotoxic effect in PAM only at concentrations exceeding 30 µM	FAN reduced replication of the virulent genotype II GZ201801 ASFV in PAM, as evidenced by decreased viral protein (p30) expression, viral B646L genome copy number, viral CP204L mRNA levels, and viral titre at both 24 and 48 hpi. The anti-ASFV activity was dose-dependent and FAN had the strongest effect in the pre-treatment mode. FAN suppressed ASFV-induced activation of the AKT/mTOR/NF-κB signaling pathway, with reduced expression of IL-1β and TNF-α in ASFV-infected PAM.	[99]
Cepharanthine (CEP)	Cytotoxic effect in PAM only at high concentrations: CC_50_ of CEP was 39.98 +/− 3.19 µM.	CEP accumulated in lysosomes and induced their alkalinization and morphological alterations, impaired endosomal trafficking, and therefore prevented proper transport and uncoating of ASFV, while also suppressing viral DNA synthesis and the formation of viral replication factories.	[100]

MOI: multiplicity of infection; CC_50_: half maximal cytotoxic concentration; PAM: porcine alveolar macrophages; moMΦ: monocyte-derived macrophages.

## 5. Conclusions and Future Strategies

The rapid global spread of ASFV is heavily exacerbated by the current lack of safe and efficient vaccines. Consequently, a targeted modulation of the host immune system is crucial to design effective therapeutic strategies against this virus. In this review, we summarize the current knowledge on immunomodulators against ASFV. As stated above, macrophages are the primary target cells of the virus, and virulent strains have developed sophisticated mechanisms to impair their antiviral activities, facilitating efficient viral replication. To counteract this subversion, several researchers have investigated macrophage-directed approaches to restore cellular defenses. In particular, the use of whole bacteria or their derivatives as immunomodulators could assist host macrophages in reprogramming their functional pathways, thereby restoring both antiviral and phagocytic capacities.

Regarding bacterial immunomodulators, some innovative attempts have recently been made. Among them, the heat-inactivated *R. nasimurium* successfully polarized porcine macrophages toward a pro-inflammatory and antiviral phenotype, demonstrating the ability to inhibit ASFV replication *in vitro* [34]. Nevertheless, when this inactivated bacterium was used as a vaccine adjuvant co-administered with the ASF BA71ΔCD2 LAV, it compromised vaccine efficacy, resulting in lower protection against challenge with a virulent ASFV isolate. This unexpected effect was likely due to the adjuvant-induced antiviral state, which restricted LAV replication in macrophages, with subsequent impairment of its capacity to trigger the development of a protective immune response [35]. These findings suggest that the association of bacterial adjuvants with ASFV-based LAVs might follow different rules depending on the baseline attenuation of the vaccine candidate. On one hand, such adjuvants could be highly effective when combined with less attenuated LAVs that still induce significant side effects, as the adjuvant would help control excessive viral replication and clinical pathology. On the other hand, they might inadvertently compromise the potency of already well-attenuated candidates.

Another crucial aspect is the characterization of the microbiota of African wild suids, such as bush pigs and warthogs, which are resistant to the disease despite some level of viral replication [17]. *R. nasimurium* was indeed isolated from warthog fecal microbiota, and studying the microbiome of resistant individuals would significantly accelerate the discovery of novel and effective adjuvants against ASFV. In addition, the host microbiota deeply shapes the baseline immune response to pathogens; therefore, its comprehensive characterization is fundamental not only for identifying new immunomodulatory components, but also for understanding how it might influence vaccine responses in domestic animals under field conditions. Future studies should investigate whether it is crucial to achieve animal eubiosis before vaccination with LAVs, in order to prevent hyper-inflammatory responses and maximize the development of a protective immune response against the virus.

In addition to whole-bacteria strategies, this review also addresses the immunomodulatory properties of diverse TLR- agonists and evaluates their potential implementation as targeted vaccine adjuvants against ASFV. We observed that a TLR-2 agonist (Mag-Pam2Cys_P48) polarized porcine macrophages toward a pro-inflammatory and anti-viral phenotype, but surprisingly it did not restrict the replication of either the attenuated NH/P68 ASFV or the virulent 26544/OG10 strain within these cells. Stimulation with Mag-Pam2Cys_P48 promoted the release of pro-inflammatory cytokines in response to ASFV infection. However, because this synthetic lipopeptide enhanced virulent ASFV replication, it may have limited applicability as a prophylactic or immunopotentiator agent against virulent ASFV strains [45]. Nevertheless, unlike *R. nasimurium*, which induced a strict antiviral state that compromised LAV efficacy [35], this synthetic lipopeptide might work in an opposite way. Specifically, Mag-Pam2Cys_P48 might enhance replication of highly attenuated LAV candidates that are currently safe but suffer from poor immunogenicity. It can provide a ‘danger signal’ that would trigger the development of a protective immune response against these LAVs. In addition, it might represent a useful adjuvant for inactivated or subunit vaccines.

Recent data suggest that TLR-4 activation might similarly enhance ASFV replication, raising the question of whether ASFV thrives more efficiently within a pro-inflammatory microenvironment. This aspect requires deeper investigation. Concerning intracellular TLR-s, the virus possesses numerous mechanisms to inhibit TLR-3 and TLR-8/9 [78], suggesting that targeting these pathways could be highly beneficial. Furthermore, the stimulation of TLR-3 and TLR-8/9 induces a milder inflammatory response alongside a higher release of type I IFNs, which effectively inhibits viral replication, suggesting that the effects of synthetic ligands for these TLR-s would improve the host’s capacity to respond to ASFV. Nevertheless, the available literature shows that complex mechanisms come into play in vivo; thus, the use of these compounds can potentially make an attenuated vaccine candidate less effective than expected. Hence, more studies are required to fully understand these networks and thus design more effective vaccines.

As for the other highlighted molecules that successfully inhibit replication *in vitro*, such as tetrandrine, berbamine, and fangchinoline, how can we implement them *in vivo*? These molecules might be used as a direct metaphylactic treatment during field outbreaks. The early administration to exposed or contact animals could establish a localized antiviral and anti-inflammatory state, potentially blocking viral entry and shedding, thus slowing down transmission within the herd.

Although providing a comprehensive review on the complex network of interactions of the immune-modulators acting against ASFV, the main limitation of this work is that the majority of the available literature on immunomodulators against ASFV relies on *in vitro* experiments. In this view, further *in vivo* studies are desirable to fully support and understand the impact of these immunomodulators on immune responses against ASFV, besides enabling the accurate evaluation of their use as vaccine adjuvants and/or their integration into immunization protocols against highly virulent ASFV strains in field conditions. Another critical factor that must be considered is that the cost of the different immunomodulators was not addressed in the available literature. A potential limitation for the large-scale use of some of the tested molecules in the field might be their high production costs, which must be carefully evaluated when designing specific prophylactic or therapeutic strategies.

Overall, in this review we summarized the current knowledge on host-directed immunomodulatory approaches against ASFV. We reported that most evidence is currently limited to *in vitro* models, and further *in vivo* studies are required to translate these preliminary results into practical application in the field. Nevertheless, a targeted modulation of the host immune system could potentially improve the design of future vaccines or treatments against this virus, to decrease viral replication without compromising vaccine efficacy. Therefore, a better understanding of the immune-modulatory properties of several molecules may contribute to the design of safe and effective vaccines or treatments against this fatal hemorrhagic viral disease.

## Data Availability

No new data were created or analyzed in this study. Data sharing is not applicable to this article.
